# Newly learned items form long-term memory in severe Alzheimer’s disease despite hippocampal atrophy

**DOI:** 10.1093/braincomms/fcag399

**Published:** 2026-10-21

**Authors:** Clément Vesque, Shailendra Segobin, Renaud Coppalle, Amy Colas, Mikaël Naveau, Caroline Mauger, Marion Hommet, Vincent de la Sayette, Francis Eustache, Hervé Platel, Mathilde Groussard

**Affiliations:** Université de Caen Normandie, Inserm, EPHE-PSL, PSL Université Paris, CHU de Caen, GIP Cyceron, U1077, NIMH, 14000 Caen, France; Université de Caen Normandie, Inserm, EPHE-PSL, PSL Université Paris, CHU de Caen, GIP Cyceron, U1077, NIMH, 14000 Caen, France; Université de Caen Normandie, Inserm, EPHE-PSL, PSL Université Paris, CHU de Caen, GIP Cyceron, U1077, NIMH, 14000 Caen, France; CRP-CPO - Centre de Recherche en Psychologie: Cognition, Psychisme et Organisations - UR UPJV 7273, 80025 Amiens, France; Université de Caen Normandie, Inserm, EPHE-PSL, PSL Université Paris, CHU de Caen, GIP Cyceron, U1077, NIMH, 14000 Caen, France; Université de Caen Normandie, Normandie Univ, CNRS, INSERM, UAR3408 US50, GIP Cyceron, 14000 Caen, France; Université de Caen Normandie, Inserm, EPHE-PSL, PSL Université Paris, CHU de Caen, GIP Cyceron, U1077, NIMH, 14000 Caen, France; Université de Caen Normandie, Inserm, EPHE-PSL, PSL Université Paris, CHU de Caen, GIP Cyceron, U1077, NIMH, 14000 Caen, France; Université de Caen Normandie, Inserm, EPHE-PSL, PSL Université Paris, CHU de Caen, GIP Cyceron, U1077, NIMH, 14000 Caen, France; Université de Caen Normandie, Inserm, EPHE-PSL, PSL Université Paris, CHU de Caen, GIP Cyceron, U1077, NIMH, 14000 Caen, France; Université de Caen Normandie, Inserm, EPHE-PSL, PSL Université Paris, CHU de Caen, GIP Cyceron, U1077, NIMH, 14000 Caen, France; Université de Caen Normandie, Inserm, EPHE-PSL, PSL Université Paris, CHU de Caen, GIP Cyceron, U1077, NIMH, 14000 Caen, France

**Keywords:** neurodegeneration, recognition, voxel-based morphometry, hippocampus, music

## Abstract

Alzheimer’s disease is a neurodegenerative disorder marked by a progressive decline in episodic memory and impaired encoding of new information. While hippocampal atrophy is a hallmark of the disease, several observations suggest that some mechanisms may remain preserved, including familiarity-based recognition. Artistic items, such as music and paintings, are particularly suitable to elicit these spared processes, as they engage reward, emotional, and implicit learning networks that remain intact in this condition. However, the extent to which patients with moderate to severe Alzheimer’s disease can learn new information in these domains remains unclear. Thirty-three patients with Alzheimer’s disease (79.30 ± 5.82 years; mean Mini-Mental State Examination [MMSE] score = 14.76 ± 5.42) and 24 healthy control participants (77.17 ± 4.62 years; mean MMSE score = 28.83 ± 0.92) were included in the protocol. A data-driven stratification based on neuropsychological scores identified two subgroups of patients, classified as moderate and severe cognitive impairments. Participants first completed repeated exposure sessions to unknown musical and pictorial items until they reported a sense of familiarity, assessed by a six-point scale designed for this population. One to two days later, a recognition session during which target items and distractor items were presented was conducted; participants’ performance was also evaluated using the familiarity scale. Structural MRI was acquired to quantify hippocampal atrophy and examine the relationship between grey matter volume and recognition performance. Despite significantly different learning rates across clinical groups (cumulative link mixed model, all pairwise comparisons *P* < 0.001), 31 patients out of 33 reached the learning criterion for at least one of the two artistic materials. In the recognition session, pairwise comparisons confirmed that all clinical groups significantly discriminated target from distractor items (cumulative link mixed model, all pairwise comparisons *P* < 0.001), with a gradient of discrimination consistent with disease severity, indicating preserved but modulated familiarity-based recognition. Structural imaging confirmed substantial hippocampal atrophy in both patient subgroups relative to healthy controls (*P* < 0.001). Multiple regression revealed that higher familiarity scores in recognition session were associated with greater grey matter volume in the right caudate nucleus (cluster-level, *P*_FWE_ < 0.05). These findings demonstrate that patients with moderate to severe Alzheimer’s disease retain the ability to encode new musical and pictorial information despite pronounced hippocampal atrophy. Familiarity-based mechanisms, supported by subcortical regions, appear to underpin this preserved learning and may offer promising perspectives for care in patients with Alzheimer’s disease.

## Teaser

Vesque *et al.* report that patients with moderate-to-severe Alzheimer's disease retain the ability to acquire familiarity for novel artistic items through repeated exposure, despite hippocampal atrophy. Recognition was preserved across all clinical groups and grey matter density in the caudate nucleus was positively associated with recognition performance for musical items.

## Graphical Abstract

**Graphical Abstract:**
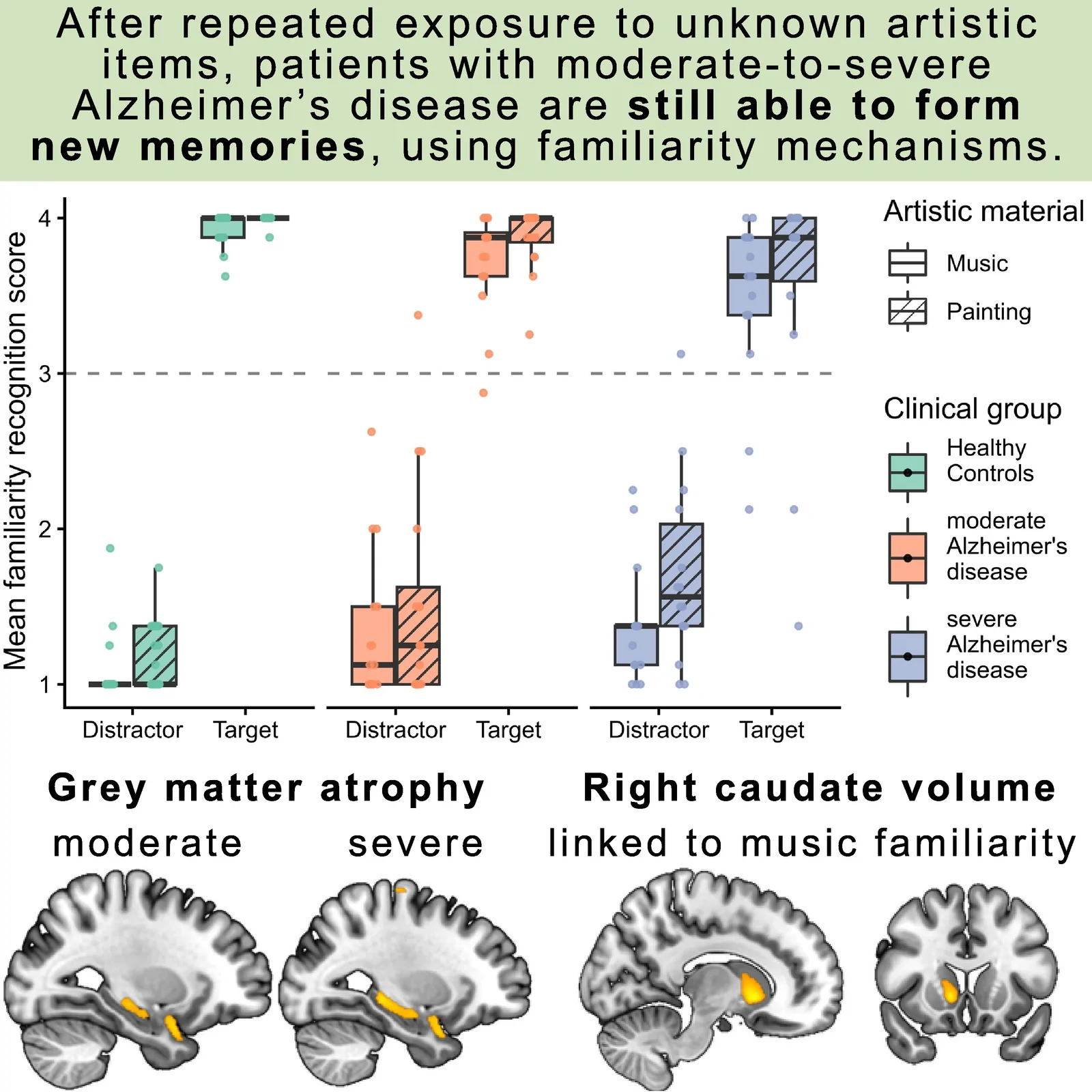

## Introduction

Dementia is a clinical label denoting a broad spectrum of cognitive impairments, resulting in significant functional deterioration in daily living, including domestic life and social engagement.^1^ As reported by the World Health Organization, 57 million people worldwide were affected by dementia in 2021, with nearly 10 million new cases emerging each year. Alzheimer’s disease accounts for 60–70% of these cases.^2^ While amyloid and tau pathologies remain its hallmark features, recent work highlights that Alzheimer’s disease is better understood as a multifactorial disorder. Thus, neuroinflammation, cerebrovascular pathologies and genetic factors could contribute alongside amyloid deposition to drive cerebral atrophy, most pronounced in the hippocampus, entorhinal cortex and neocortex.^3,4^ These structural changes result in severe impairment of declarative memory, affecting both the retrieval of existing information and the encoding of new information into long-term memory.^5,6^

Yet, despite these profound impairments, some forms of implicit memory seem to be mostly functional for patients with Alzheimer’s disease up until moderate to severe stages. To illustrate specific implicit memory preservation, numerous articles have revealed preserved mere exposure effect—which is increased preference for repeatedly exposed items^7,8^—contrasting with altered recognition in patients with Alzheimer’s disease from a mild stage.^9-12^ This dissociation strongly suggests functional independence between implicit and explicit forms of memory, and occurs following damages specifically targeting the entorhinal and perirhinal cortices in the medial temporal lobe.^13-15^ Moreover, another type of successful long-term new encoding has been uncovered using paradigms developed to provide errorless learning. Thus, in procedural tasks, patients with mild to moderate Alzheimer’s disease are still able to learn, using techniques such as spaced retrieval (i.e. ability to repeatedly recall newly acquired information over increasingly longer time periods) or vanishing cues (i.e. recall attempts using cues, that are gradually reduced until successful recall, without any mistakes).^16^ Errorless learning (while used in patients with Alzheimer’s disease at early stages) showed promising results but mostly in regaining the possibility to learn daily tasks, especially with the help of previous semantic knowledge as shown with single-case studies.^17,18^ However, although encoding of some procedures using these techniques is possible in Alzheimer’s disease patients, the performance outcome remains low,^19^ and decreases over the course of the disease, to become null at late stages.^17^ The possibility of new long-term encoding in anterograde amnesia has also been studied in other pathologies involving severe damage to the medial temporal lobe besides Alzheimer’s disease. Until the late 1990s, there was a large debate about the required involvement of the hippocampus (and more broadly the medial temporal lobe) in learning semantic information.^20,21^ Although H.M.’s famous case of amnesia^22^ constituted the main evidence of the involvement of medial temporal lobe for encoding in long-term memory, Vargha-Khadem *et al.*^23^ studies on developmental amnesia resulting in hippocampal atrophy showed that the acquisition of new semantic knowledge is still possible even with severe focal or more extended hippocampal damages.

However, explicit memory itself is not a unitary system, but rather encompasses both recollection and familiarity. Recollection process is linked to episodic memory and heavily dependent on the integrity of the hippocampus, while familiarity, more closely tied to the semantic memory, relies on the integrity of the perirhinal cortex.^6,24^ More specifically, familiarity-based recognition (FBR) can be defined as the ability to recognize a previously encountered stimulus as familiar, even in the absence of contextual recollection. In other words, it is a feeling of ‘knowing’ something without ‘remembering’ it.^25,26^ Indeed, one puzzling dissociation between familiarity and recollection was brought by Bowles *et al.*^27^ They reported that N.B., a patient with perirhinal and amygdala damages in the left hemisphere but no hippocampal damages, showed severely impaired FBR but normal recollection-based recognition. Given that these structures are not equally affected in Alzheimer’s disease, FBR may remain partially preserved, even as recollection deteriorates. From both remote and recent work on FBR, a growing body of studies advocates for the possibility to use familiarity to encode and retrieve semantic information with extensive hippocampal damage.^22,23,28^ Paradoxically, whilst these mechanisms suggest that FBR may be preserved, patients with Alzheimer’s disease are generally considered unable to rely on FBR at a late stage,^29^ and even at an early stage,^30^ particularly because perirhinal cortex is among the first structures to be degraded in the course of Alzheimer’s disease (it is also used as an early diagnosis biomarker).^31^ Most studies concluding an absence of FBR have used verbal or visual material that engages networks affected early on by the disease.^13,29,30,32^

However, isolated observations suggest the opposite, particularly when using auditory material. Indeed, musical material appears particularly well-suited to elicit preserved memory processes in Alzheimer’s disease, considering that musical abilities and memory for old tunes are very well spared in patients with Alzheimer’s disease even at a late stage.^33^ For instance, Quoniam *et al.*^34^ demonstrated that even when explicit recognition was strongly impaired, patients showed a graded mere exposure with musical material, depending on the number of presentations. Other studies using music with different designs confirmed the dissociation between functional mere exposure effect and altered explicit memory for patients with Alzheimer’s disease at a mild stage.^6,29,35^ More specifically, Samson *et al.* demonstrated that patients with moderate to severe Alzheimer’s disease had a stronger sense of familiarity (SoF)—the subjective feeling of having previously encountered a stimulus, with weak confidence—with musical items than with verbal items.^36^ Similarly, Coppalle *et al.*^37^ demonstrated that learning was still possible for patients at this stage. However, this appears to be modulated by the nature of the items, with visual and auditory artistic items (i.e. painting and music) eliciting a greater SoF, especially for music items. The study reports that, during the tests, patients were more easily engaged by music than by paintings. This evidence regarding the preservation of SoF in this population for musical items leads us to question the neurobiological substrates that may underlie it.

Indeed, like explicit memory, musical memory can be divided into semantic musical memory and episodic musical memory, with each relying on distinct cerebral substrates. Thus, semantic musical memory was supported by frontal and temporal regions, while episodic musical memory was supported by the precuneus, medial frontal cortex and superior frontal gyrus.^38,39^ Using PET-FDG and MRI scans in Alzheimer’s disease patients, Jacobsen *et al.* have shown that there is no overlap between cortical degeneration, glucose hypometabolism (both inherent to the pathology) and the anterior cingulate cortex (ACC) and the ventral pre-supplementary motor area (pre-SMA).^40^ The ACC is well known to be involved in familiarity,^41^ while pre-SMA is also involved in planification and attentional switching.^42^ As these regions associated with musical memory show the least grey matter (GM) atrophy and hypometabolism, these findings suggest that the maintenance of musical semantic memory, which underlies musical FBR, is facilitated by the preservation of these regions. Furthermore, the ACC belongs to the salience network, which is engaged in response to novel or surprising stimuli associated with musical listening.^43-45^ This network is well known to be linked to dopaminergic activation and to be activated by music.^45^ Consistent with this, King *et al.* showed that musical listening induced a temporary increase in connectivity within this network in Alzheimer’s disease patients, which could potentially account for transient improvements in memory symptoms.^43^ It suggests that the salience network remains relatively intact in Alzheimer’s disease. Finally, Benhamou *et al.* demonstrated that patients with Alzheimer’s disease exhibited normal reactivity to deviance in familiar musical excerpts, suggesting that the central processing of musical attention remains functional.^46^

Despite growing interest in the role of music in Alzheimer’s disease care, existing studies have focused predominantly on early and mild stages of the disease, rarely including neuroanatomical data and remain limited in sample size. Until now, the ability of Alzheimer’s patients in the moderate to severe stages to acquire new artistic knowledge based on SoF has not been studied. It is in this context that the INCAS protocol (Imaging of New Knowledge in Severe Alzheimer) was developed.

First, we aimed to determine whether patients with Alzheimer’s disease—despite severe episodic amnesia—can still experience FBR for artistic items (music and paintings), comparable to that of healthy controls. We predicted that repeated exposure would enable patients to acquire familiarity for artistic items, even at advanced disease stages. Then, we assessed whether both healthy controls and patients were able to discriminate targets from distractors, hypothesizing that this discrimination would be preserved regardless of disease severity. We also explored whether the nature of the artistic material (music and paintings) modulated SoF acquisition and recognition performance. Finally, after quantifying cerebral and hippocampal atrophy, we examined the relationship between recognition performance and GM atrophy, hypothesizing that GM volume would be positively correlated with familiarity scores in the recognition session, with potential contributions from preserved cortical and subcortical regions.

## Materials and methods

### Population

A total of 33 patients with Alzheimer's disease (79.30 ± 5.82 years; Mini-Mental State Examination [MMSE] = 14.76 ± 5.42 [3.00–25.00]) and 24 healthy participants (health controls [HC]; 77.17 ± 4.62 years; MMSE = 28.83 ± 0.92 [26.00–30.00]) were recruited. All participants were over 60 years old, spoke French as their first language, and had no neurological, psychiatric or cardiac history, nor MRI contraindications. None were expert musicians^47^ or played music regularly; all completed an artistic culture questionnaire assessing past and current involvement in music or painting, as well as interest and knowledge in these domains. All participants, along with their informal caregivers for patients with Alzheimer's disease if applicable, gave written consent for inclusion in this protocol. This study was approved by an ethics committee (CPP Nord-Ouest III, ID-RCB n°2011-A00351-40) and conducted in accordance with the Declaration of Helsinki.

For the HC group, participants showed healthy ageing, that is, they had a score considered to be within the norm on the MMSE,^48^ the MATTIS Dementia Rating Scale^49^ and a score equal to zero on the Clinical Dementia Rating (CDR).^50^ Participants had no sleep disorders (Pittsburgh Sleep Quality Index [PSQI] ≤ 10)^51^ or depressive symptoms (Geriatric Depression Scale [GDS] < 10)^52^ and reported no memory complaints.

Patients were recruited through a partnership with independent living facilities in the Normandy region of France. The inclusion criteria encompassed meeting the NINCDS-ADRDA Alzheimer's Diagnosis Criteria and having a probable Alzheimer's disease diagnosis from a neurologist or geriatrician prior to the beginning of the study. A data-driven classification distinguished moderate Alzheimer’s Disease (mAD) and severe Alzheimer’s Disease (sAD) subgroups, based on their neuropsychological scores (Supplementary Fig. 1). Therefore, the three groups that were formed in our paradigm are hereafter referred to as HC, mAD and sAD. Patients’ cognitive profiles were assessed using global cognition (MMSE,^48^ BEC96 battery test),^53^ disease severity (CDR),^50^ lexical access (80-images oral naming test; DO80)^54^ and verbal episodic memory (five words of Dubois test).^55^

### Materials

#### Scale of familiarity

Conventional recognition paradigms relying on ‘Yes/No’ responses often fail to account for learning in Alzheimer’s disease patients. Indeed, they may show either great hesitation or language impairment at a moderate to severe stage, which results in difficulties accounting for answers based solely on verbal answers. For this reason, behavioural cues must be considered when measuring changes in behaviour that reflect effective modifications to indicators of familiarity. Therefore, the SoF Scale was developed by our team to specifically assess the level of recognition of individuals with amnesia (Table 1).^36,37^ Thus, following the presentation of each item, participants were asked to what extent they felt familiar with it. Scores were attributed by the examiner based on verbal answers (‘I don’t know this music’, ‘Maybe I’ve already seen that’, ‘Yes, I heard that yesterday’) and behavioural reactions (hesitations, wrinkled forehead and eyes). Based on these cues, a score ranging from one to six is established, as detailed in Table 1. Scores one and two indicate an absence of any form of conscious recognition and, respectively, without or with marks of interest. These scores are indicative of a complete absence of familiarity by the participant. A score of three indicates a degree of weak familiarity, or the emergence of an SoF. This score is characterized by hesitation in responding or an absence of any feeling of déjà vu for the item. A score of four denotes a confident recognition of the item, but no recall of the context in which it was learned. Finally, a score of five or six suggests recollection—when individuals provide more specific information about the context. A score of six is associated with accurate episodic memory. Given the severity of cognitive impairment in our sample, the threshold for familiarity acquisition was set at a score of three or higher, capturing the emergence of SoF. Statistical analyses of the exposure and recognition sessions focused on scores of four or lower, corresponding to FBR. Then, scores of five and six were recoded as four to prioritize FBR over recollection. Answers from the participants were collected by an examiner specializing in neuropsychology and coded using this scale, with the rules described earlier. The scoring was carried out immediately or later by reviewing the video recording of the session. In case of doubt regarding the score to be awarded, another psychologist was consulted to confirm or refute the score.

**Table 1:** Sense of familiarity (SoF) Scale

Familiarity Score	Behavioural response	Example of verbal answer	Memory process
1	No recognition or interest	‘Never heard it before in my life…’	None
2	No recognition but signs of interest	‘I don’t know this one but it’s pretty’	Mere exposure effect
3	Emergence of the SoF, uncertainty	‘I feel like I have heard that before, but I am not quite sure…’	Sense of Familiarity (SoF)
4	Recognition, wrong or incorrect context	‘Oh, yes, I like this one but where did that come from? Maybe I heard it with my friend?’	Familiarity-based recognition (FBR)
5	Recognition with imprecise context	‘I’m sure I’ve seen this not that long ago…Maybe with you?’	Weak recollection
6	Recognition with rich context	‘Yes, we listened to it together, last week with other tunes and paintings. Then, you asked me the same questions!’	Recollection

SoF: Sense of Familiarity. Adapted from Coppalle *et al.*^37^.

#### Artistic materials

According to previous studies on Alzheimer’s disease, we decided to integrate music and painting as artistic items. Indeed, these kinds of artistic items appear to be particularly relevant for our population to reveal memory processes because they seem to evoke a stronger SoF scale score than verbal items, such as poems.^33,37^ A total of 32 pieces have been selected for the study, including 16 real musical compositions and 16 real paintings. The duration of each stimulus presentation ranged from 10 to 20 s. The musical pieces presented were classified as either pop-folk music or French varieties with lyrics and performed by a single singer with a dominant instrument. Regarding the paintings, they were all figurative and classified as naturalistic, impressionist, classicist or humanist painting. The selection of items was reviewed by a cohort of 28 healthy individuals aged 60 years and over to ensure that the items were not previously known to them. In both artistic domains, half of these items were designated as targets and were subsequently presented to them. The remaining items were designated as distractors, to assess recognition without prior exposure. Consequently, the musical material comprised eight targets and eight distractors, as did the pictorial material.

### Procedure

#### Learning paradigm

We conducted individual exposure sessions, consisting of a single presentation of every item: eight musical excerpts and eight paintings (Fig. 1). The order of the artistic categories was randomized, and items were presented in a random order within each category. The objective of these sessions was to assess the emergence of the SoF between the beginning of the exposure sessions and their end, depending on the artistic material and the clinical status. Items presented in these sessions were the target items and referred to unfamiliar music or paintings. They were presented in a pseudo-randomized order and the order was changed for every participant to avoid serial position effects. During each session, participants were merely encouraged to comment on the items (‘Do you feel like you have already heard/seen this?’) and no explicit instructions regarding learning or memorization were given, corresponding to incidental learning. Their knowledge of the items was assessed by the SoF scale (Table 1). The number of sessions varied between 1 and 3 for the HC group and between 1 and 10 for both AD groups. Each session lasted a maximum of 30 min. Learning was considered to have taken place when 75% or more items were rated with an SoF scale score of three or above. If this requirement was not met, a further exposure session was scheduled the next day. This procedure was repeated until the learning criterion was met, with a maximum of ten sessions allowed. Participants who failed to meet this criterion were included only in the condition in which learning criterion was met. For instance, participants who met the criterion for music but not for painting were included only in the statistical analyses related to music condition and vice versa.

**Figure 1:**
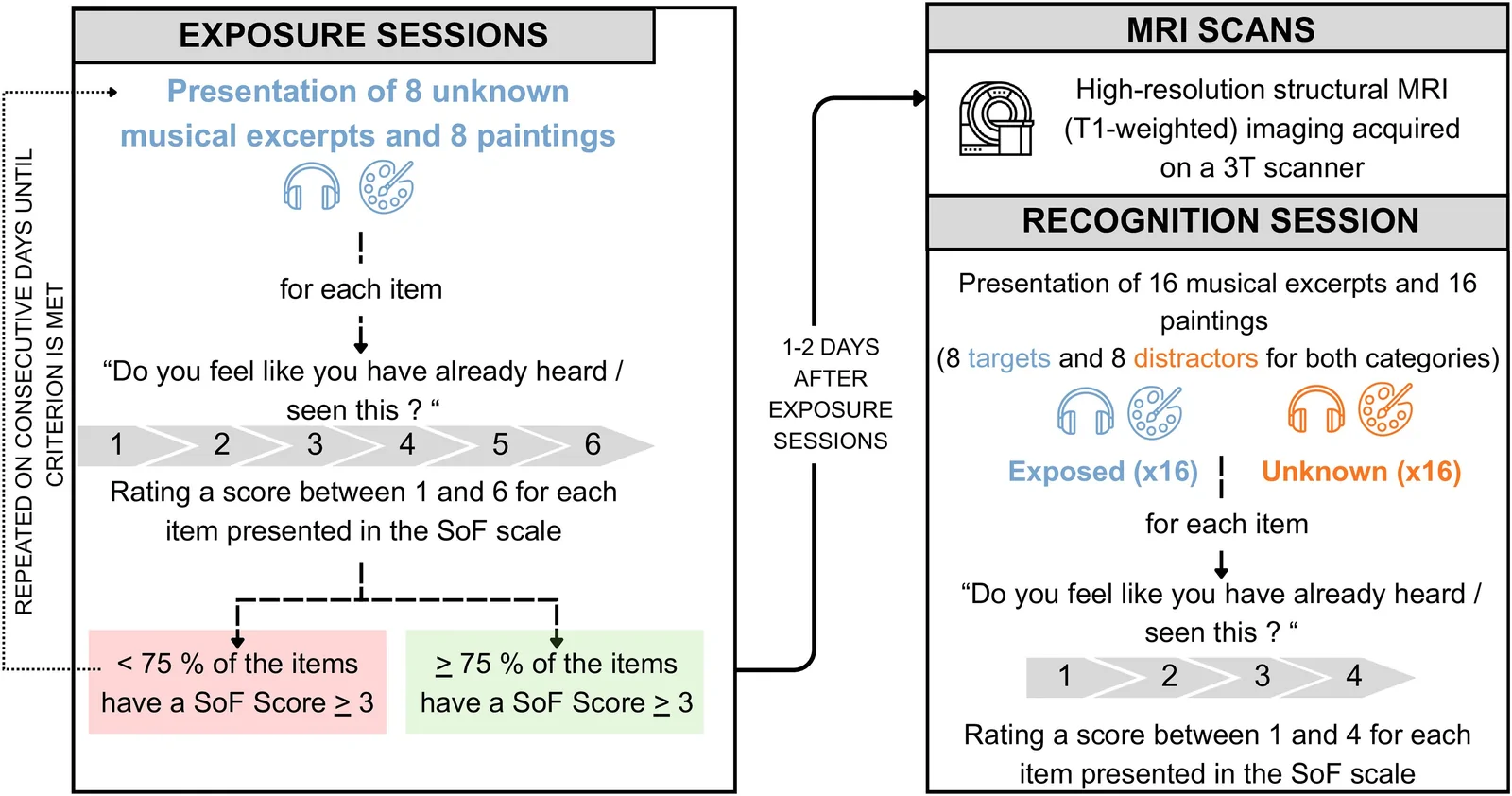
**Behavioural experiment design.** Coloured dashed box indicates the learning criterion. SoF scale: Sense of Familiarity scale. Scores 5 and 6 were recoded as 4 for recognition analyses.

Upon completion of the exposure sessions, participants underwent a recognition session the following day (from 1 to 2 days). Only participants who reached the learning criterion were given this task. Due to overwhelming anxiety, one HC participant was unable to attend the recognition session for both artistic material; one mAD participant was unable to attend it for music only and another mAD participant was unable to attend it for painting only; one sAD participant was unable to attend it for painting only. The exclusion of these subjects was taken into consideration during the final determination of the sample size for the statistical analysis of the recognition session. During this session, the targets, presented in the exposure sessions, and the distractors, not presented before and not notoriously known, were randomly presented. Participants were simply prompted to comment on the items and asked a question about their familiarity with them. Their answers were evaluated using the SoF scale.

#### MRI acquisition and pre-processing

To assess the extent of brain atrophy and, more specifically, confirm the hippocampal atrophy typically observed in patients, anatomical MRI scans were conducted. A high-resolution T1-weighted image was acquired for each subject on a Philips Achieva 3T scanner using a 3D fast-field echo sequence (sagittal; repetition time = 20 ms; echo time = 4.6 ms; flip angle = 10^°^; 180 slices; slice thickness, 1 mm; field of view, 256 × 256 mm^2^; matrix, 256 × 256). Imaging data were pre-processed using fMRIPrep version 25.0.0, a standardized pipeline based on Nipype,^56^ including spatial normalization to the OASIS30ANTs template (MNI152NLin2009cAsym space, 1 × 1 × 1 mm^3^), specifically suited for aged and atrophied brains. T1-weighted images were subsequently processed using the *SPM12* toolbox (https://www.fil.ion.ucl.ac.uk/spm/software/Statistical Parametric Mapping software; Wellcome Department of Cognitive Neurology, Institute of Neurology, London, UK), including segmentation into GM, white matter (WM) and cerebrospinal fluid (CSF) and modulation to preserve local GM volume. Modulated GM images were smoothed with an 8 mm full-width at half-maximum (FWHM) isotropic Gaussian kernel for voxel-based morphometry (VBM) analyses. Non-modulated and non-smoothed GM images from healthy controls were averaged (threshold at 0.5) to create a GM mask for statistical analyses. A total intracranial volume (TIV) was calculated based on the sum of the total individual volumes of GM, WM and CSF for each participant.

### Statistical analysis

The statistical analyses and the corresponding graphs were performed using the *RStudio* software (version 4.4.2) and *SPM12* for the MRI examinations. A significance level of *α* = 0.05 was used for tests. Cerebral representations were obtained using *MRIcroGL* (version 13.7.8).

#### Neuropsychological assessments

Due to the non-compliance with the normality assumption (as verified through a Shapiro–Wilk test) and the limited sample size, a Kruskal–Wallis’ test was performed between the three groups of clinical statuses for inter-group comparisons. If a discrepancy was identified, a Dunn’s *post hoc* test, adjusted for Holm’s correction, was employed. Effect size was calculated using the Kruskal–Wallis’ eta squared.

#### Learning paradigm

For exposure sessions, we analysed SoF scale score obtained during exposure sessions to track the learning progress of the three participant groups (HC; mAD; sAD). A score ranging from one to six was given for each session and for each item presented. Scores five and six were recoded as four to study the FBR specifically. Given the ordinal nature of the SoF scale score, the longitudinal structure of the data and to model learning trajectories across sessions, we employed cumulative link mixed models *(clmm)* implemented into the R package *ordinal.*^57^ This approach is adapted for repeated-measures designs involving ordinal outcome variables and does not assume normality or equal intervals between scale categories. Furthermore, this strategy encompassed random effects, such as inter-item and inter-participant variability. The session number was treated as a continuous variable to account for the structurally imbalanced design, in which HC participants completed fewer sessions than the patient groups. Subsequently, three models were specified. The first model (m1_exposure) incorporated ‘Session’, ‘Clinical Status’ and ‘Artistic Material’ as additive fixed effects, assuming identical learning slopes across groups. The second model (m2_exposure) added a ‘Session × Clinical status’ interaction, with ‘Artistic material’, allowing learning slopes to differ between clinical groups. The third model (m3_exposure) retained the ‘Session × Clinical status’ interaction but excluded ‘Artistic Material’, to assess whether this variable contributed meaningfully to model fit. The models were fitted using a symmetric threshold parameterization. Using likelihood ratio tests (LRT), we first compared m1_exposure and m2_exposure to assess whether the addition of the ‘Session × Clinical status’ interaction significantly improved model fit. We then compared m2_exposure and m3_exposure to determine whether the inclusion of artistic material significantly improved model fit. The model with the best fit was retained for further analyses. Pairwise comparisons of estimated marginal mean SoF scale scores between clinical status were then performed within the retained model using Tukey-adjusted contrasts from the *emmeans* package.^58^ Following this, learning slopes for each clinical group were estimated from the best model, using the *emtrends* function and pairwise comparisons between slopes were performed using Tukey correction. Fixed-effect estimates from the best model are reported as log-odds coefficients (β) with their standard errors. To facilitate biological interpretation, log-odds coefficients were exponentiated to obtain odds ratios, reflecting the multiplicative change in the odds of obtaining a higher SoF scale score per additional session for each clinical group. Effect sizes were calculated using the Cohen’s *r.*

For the recognition task, we analysed SoF scale score obtained after the presentation of ‘Target’ item (presented during exposure sessions) and ‘Distractor’ item (not presented during exposure sessions). Only participants who reached the learning criterion in exposure sessions were included in these analyses. Thirty-two items were presented for each participant: eight music and eight paintings for both targets and distractors. As with the exposure sessions, cumulative link mixed models were used to analyse these data. The first model (m1_recognition) incorporated ‘Type’, ‘Clinical status’ and ‘Artistic Material’ as additive fixed effects, assuming identical recognition scores across groups and artistic material. The second model (m2_recognition) added a ‘Type × Clinical status’ interaction, with ‘Artistic material’, allowing recognition scores to differ between groups. The third model (m3_recognition) retained the ‘Type × Clinical status’ interaction but excluded ‘Artistic Material’, to assess whether this variable contributed meaningfully to model fit. The models were fitted using a symmetric threshold parameterization. Using LRT, we first compared m1_recognition and m2_recognition to assess whether the addition of the ‘Type × Clinical status’ interaction significantly improved model fit. We then compared m2_recognition and m3_recognition to determine whether the inclusion of artistic material significantly improved model fit. The model with the best fit was retained for further analyses. Additionally, pairwise comparisons were performed separately within the retained model to assess recognition discrimination, using Tukey-adjusted contrasts from the *emmeans* package.^58^ Effect sizes were calculated using the Cohen’s *r*.

### MRI analyses

#### Whole brain atrophy

Using *SPM12*, VBM analyses were conducted to compare whole-brain GM volume across the three clinical statuses. Data were analysed using an analysis of covariance (ANCOVA) model, including age, sex and TIV as covariates. *Post hoc* pairwise comparisons between groups were performed using *t*-contrasts to compare the relative atrophy of patients compared to HC. Statistical significance was assessed using a voxel-level family-wise error correction (*P*_FWE_ < 0.05), with an extent threshold of 202.5 mm^3^ (*k* ≥ 60).^59,60^ Clusters were labelled using the AAL3v1 atlas.^61^

#### Hippocampal atrophy

Assessments of the hippocampal atrophy were carried out using *FreeSurfer's* segmentation. In addition to whole-brain VBM analyses, hippocampal volumes were extracted and compared across groups as a complementary morphometric approach. First, total bilateral hippocampal volumes were compared across the three clinical groups. Then, left and right hippocampal volumes were analysed separately to assess potential hemispheric differences. Hippocampal volumes were first adjusted for age, sex and TIV using a linear regression model. Group differences were assessed on the adjusted values using a Kruskal–Wallis test, followed by post-hoc pairwise comparisons using Dunn’s tests with Holm’s correction. Effect size was calculated using the Kruskal–Wallis’ eta squared. Finally, intra-groups comparisons of hippocampal volumes were assessed using Wilcoxon tests. Effect size was calculated using the rank biserial *r*.

#### Multiple regression between the SoF and the grey matter volume

Finally, to assess whether SoF were associated with specific brain regions, multiple regression analysis was performed, using SoF scale score at the recognition session as the variable of interest and GM volume as the neuroimaging predictor, controlling for age, sex, TIV and MMSE, to control for cognitive severity impairment. Two separate regression analyses were performed for music and painting stimuli respectively. Both analyses were performed using both mAD and sAD patients combined, in patients who meet the learning criterion in exposure session. Statistical significance was set at an uncorrected voxel-wise threshold of *P*_UNC_ <0.001, with a minimum cluster extent of *k* ≥ 60 voxels. Cluster-level inference was assessed using family-wise error correction (*P*_FWE_ < 0.05). Significant clusters were labelled using the AAL3v1 atlas.^61^

## Results

### Demographic and neuropsychological assessments

Following the classification derived from clustering analysis, a significant discrepancy (*χ*^2^ ranging from 38.55 to 50.56, all *P* < 0.001) was identified in the comparative analysis of the neuropsychological scores of HC, mAD and sAD (Table 2). Dunn’s *post hoc* comparisons with Holm’s correction revealed a significant decrease in the neuropsychological score between HC and both patient groups and between mAD and sAD.

**Table 2:** Demographic and neuropsychological characteristics

	Participants					
Characteristic	HC *N* = 24^a^	mAD *N* = 18^a^	sAD *N* = 15^a^	Statistic test	*P-value*	Effect size
Sex				χ^2^(2) = 0.51	0.775	
F	20.0 (83.3%)	16.0 (88.9%)	12.0 (80.0%)			
M	4.0 (16.7%)	2.0 (11.1%)	3.0 (20%)			
Age (years)	77.17 ± 4.62	79.39 ± 5.40	79.20 ± 6.48	χ^2^(2) = 2.47	0.290	η^2^ = 0.009
Education level (years)	9.58 ± 2.78	9.72 ± 3.77	8.93 ± 2.71	χ^2^(2) = 0.70	0.706	η^2^ = 0.000
CDR	0.00 ± 0.00	1.44 ± 0.51	2.46 ± 0.51	χ^2^(2) = 50.77	<0.001^b^	η^2^ = 0.903
MMSE	28.83 ± 0.92	18.56 ± 3.73	10.20 ± 3.08	χ^2^(2) = 49.17	<0.001^c^	η^2^ = 0.874
BEC96	91.54 ± 3.12	60.39 ± 9.73	33.00 ± 15.82	χ^2^(2) = 46.62	<0.001^d^	η^2^ = 0.826
Five-Words	9.88 ± 0.34	6.78 ± 1.63	2.20 ± 2.01	χ^2^(2) = 48.09	<0.001^e^	η^2^ = 0.854
DO80	79.29 ± 1.43	69.06 ± 9.97	51.80 ± 21.73	χ^2^(2) = 36.62	**<0**.**001**^f^	η^2^ = 0.641

BEC96, cognitive battery test; DO80, 80-images oral naming test.

Intergroup comparisons were obtained using a Kruskall–Wallis test, followed by a Dunn's *post hoc* test with Holm's correction. *P*-values are derived from independent-sample *t*-tests for continuous variables and chi-square tests for categorical variables. Significant results are displayed in bold font. *P*  *<* 0.05, *P*  *<* 0.01, *P* < 0.001. Effect size was calculated using the Kruskal–Wallis eta squared.

^a^ *n* (%); Mean ± SD.

^b^ *Post hoc* Dunn (Holm): HC versus mAD *P* < 0.001; HC versus sAD *P* < 0.001; mAD versus sAD *P* < 0.05.

^c^ *Post hoc* Dunn (Holm): HC versus mAD *P* < 0.001; HC versus sAD *P* < 0.001; mAD versus sAD *P* < 0.01.

^d^ *Post hoc* Dunn (Holm): HC versus mAD *P* < 0.001; HC versus sAD *P* < 0.001; mAD versus sAD *P* < 0.01.

^e^ *Post hoc* Dunn (Holm): HC versus mAD *P* < 0.001; HC versus sAD *P* < 0.001; mAD versus sAD *P* < 0.01.

^f^ *Post hoc* Dunn (Holm): HC versus mAD *P* < 0.001; HC versus sAD *P* < 0.001; mAD versus sAD *P* < 0.05.

### Learning paradigm

#### Exposure sessions

The number of exposure sessions required to meet the learning criterion varied across clinical groups and artistic materials, with patients’ groups requiring more sessions than HC. For the HC group, all the 24 participants successfully met the criterion for music (mean ± SD = 2.54 ± 0.51, range = 2–3) and for painting (mean ± SD = 2.42 ± 0.50, range = 2–3). For the mAD group, of the 18 participants, 17 achieved the learning criterion for music (mean ± SD = 5.18 ± 1.29, range = 4–8) and 17 for painting (mean ± SD = 5.06 ± 1.34, range = 3–8). Finally, of the 15 sAD participants, 15 achieved the learning criterion for music (mean ± SD = 6.93 ± 1.71, range = 4–10) and 15 for paintings (mean ± SD = 6.93 ± 1.79, range = 4–10).

The LRT confirmed that m2_exposure model (‘Session × Clinical status’ interaction, with ‘Artistic material’) significantly improved the model fit compared to m1_exposure model (χ^2^ (2) = 1100.5, *P*  *<* 2.2e-16). However, the inclusion of ‘Artistic material’ did not improve the model fit, compared to the model without it (χ^2^ (1) = 2.8716, *P* = 0.09015). Therefore, following the principle of parsimony, the m3_exposure model (‘Session’ × ‘Clinical status’ interaction, without ‘Artistic material’) was retained for subsequent analyses. Pairwise comparisons with Tukey-correction of estimated marginal mean SoF scale scores in m3_exposure model revealed significant differences between HC and mAD (*z* = 17.176; *P* < 0.0001, *r* = 0.21), HC and sAD (*z* = 18.496, *P* < 0.0001, *r* = 0.23) and between mAD and sAD (*z* = 2.505, *P* = 0.0328, *r* = 0.03), though the latter contrast reflected a negligible effect size. Fixed-effect estimates indicated positive learning slopes of *β*_HC_ = 6.914 ± 0.284 (OR_HC_ = 1006.27) for HC, *β*_mAD_ = 1.620 ± 0.0603 (OR_mAD_ = 5.05) for mAD and *β*_sAD_ = 0.879 ± 0.0337 (OR_sAD_ = 2.41) for sAD. Learning slopes differed significantly between all groups (HC versus mAD: *z* = 18.635, *P*  *<* 0.0001, *r* = 0.23; HC versus sAD: *z* = 21.398, *P*  *<* 0.0001*, r* = 0.27; mAD versus sAD: z = 11.372, *P*  *<* 0.0001, *r* = 0.14), consistent with preserved familiarity acquisition across all groups, despite the presence of a disease severity gradient in learning speed (Fig. 2). Taken together, these results indicate that, although the rate of learning and the overall level of SoF scale score decreased progressively with disease severity, familiarity acquisition was preserved across all clinical groups.

**Figure 2:**
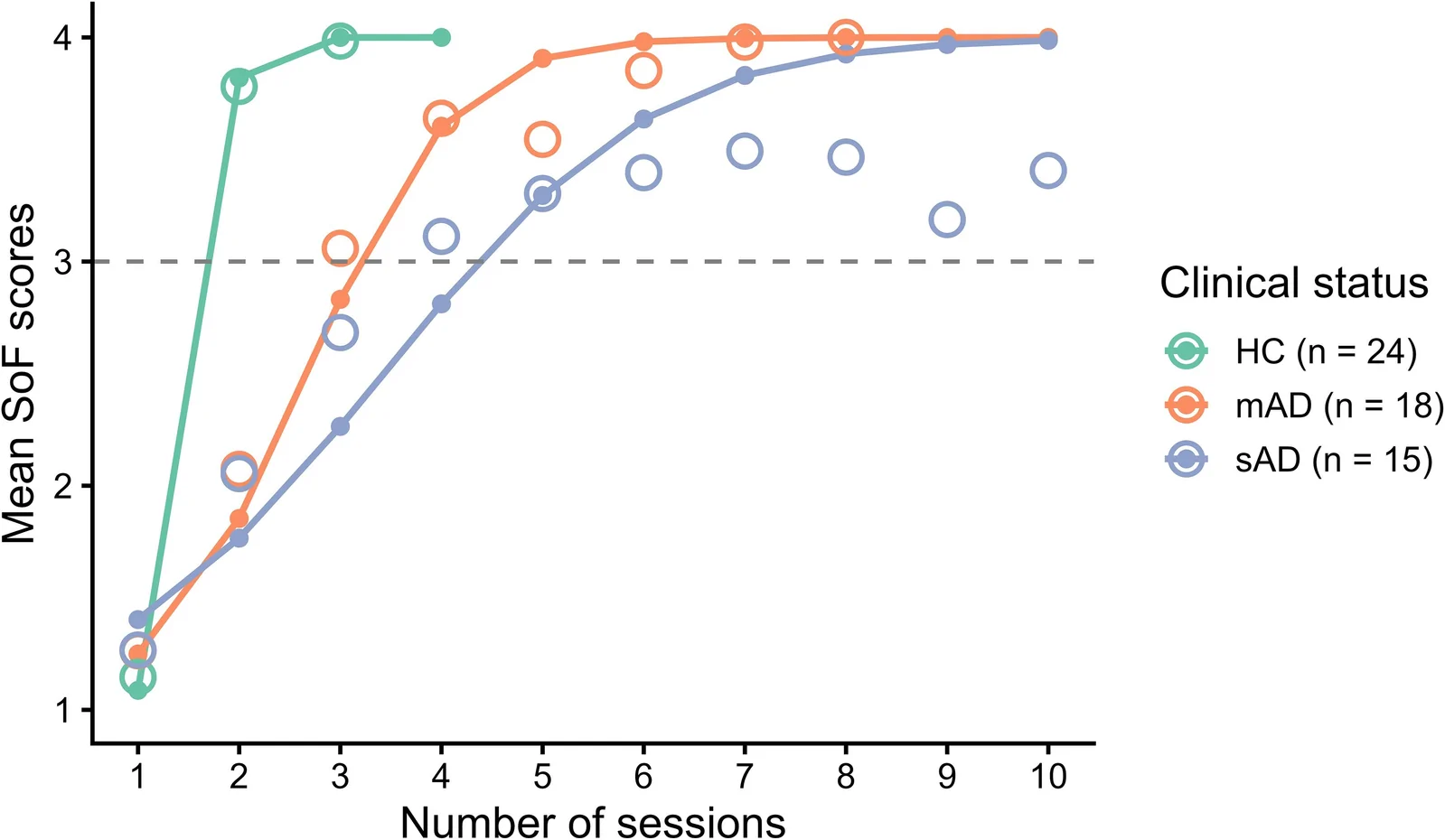
**Learning dynamics during exposure sessions.** Predicted learning trajectories derived from the cumulative link mixed model (m3_exposure) for the three clinical groups, averaged across artistic materials. *n*_HC_ = 24, *n*_mAD_ =18, *n*_sAD_ = 15 Filled dots: model-predicted values, open circles: observed group means. Dashed horizontal line indicates the SoF threshold (SoF ≥ 3). Model predictions may slightly overestimate observed scores at session 1 and at later sessions for the sAD group, reflecting a structural floor effect at baseline and progressive attrition of faster learners in later sessions, respectively. HC: healthy controls, mAD: moderate Alzheimer’s disease, sAD: severe Alzheimer’s disease.

#### Recognition session

The number of targets correctly recognized (i.e. items scoring ≥3 for targets, out of eight items per artistic material and per participant) was high across all clinical groups. Indeed, the HC group recognized an average of 7.91 ± 0.29 musical targets and 8.00 ± 0.00 pictorial targets. The mAD group recognized an average of 7.50 ± 0.89 musical targets and 7.81 ± 0.54 pictorial targets. Those with sAD recognized an average of 6.93 ± 1.53 musical targets and 7.07 ± 2.06 pictorial targets. By contrast, the number of distractors incorrectly recognized (i.e. scoring ≥3 for distractors) remained low in all three clinical groups. HC participants incorrectly recognized an average of 0.17 ± 0.49 musical distractors and 0.39 ± 0.58 pictorial distractors. The mAD participants incorrectly recognized an average of 0.88 ± 1.45 musical distractors and 1.44 ± 2.19 pictorial distractors. Finally, the sAD participants incorrectly recognized an average of 0.73 ± 1.22 musical distractors and 1.57 ± 1.74 pictorial distractors (Fig. 3). These results suggest high hit and low false alarms rates across all clinical groups, consistent with preserved FBR.

**Figure 3:**
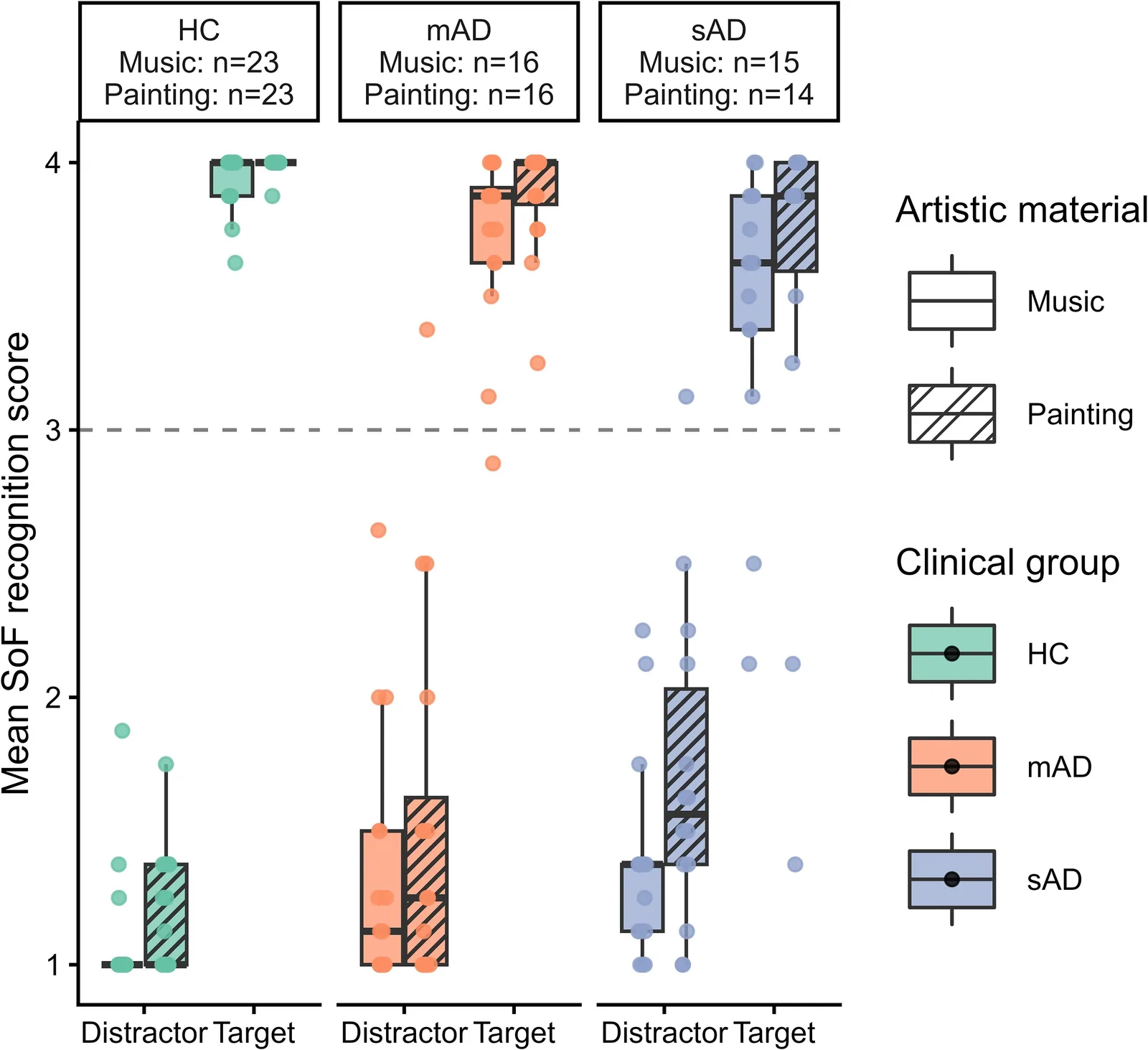
**Recognition performance across clinical groups and artistic materials.** Mean SoF recognition scores for targets (previously exposed) and distractor items (not previously exposed), for each clinical group and separated by artistic material (plain boxes = music; hatched boxes = painting). Dashed lines indicate the SoF threshold (SoF score ≥3). Dots represent individual data. Statistical analyses were performed using pairwise comparisons with Tukey-correction of estimated marginal SoF scales scores in recognition session, derived from a cumulative link mixed model (CLMM). All groups significantly discriminated targets from distractors (HC: *n* = 23, *z* = 17.873; mAD: *n* = 16, *z* = 16.903; sAD: *n* = 15 for music, *n* = 14 for painting, *z* = 14.383; all *P* < 0.0001). Between groups comparisons revealed significant differences in targets (HC versus mAD: *z* = 3.954, *P* = 0.0002; HC versus sAD: z = 6.10, *P* < 0.0001; mAD versus sAD: z = 2.514, *P* = 0.032) and in distractors (HC versus mAD: *z* = −4.425, *P* < 0.0001; HC versus sAD: *z* = −5.407, *P* < 0.0001). No significant differences were observed between mAD and sAD for distractors. Pictorial items have higher SoF scores than musical items across all groups and items types (music versus painting: z = 3.339, *P* = 0.0008). HC: healthy controls; mAD: moderate Alzheimer’s disease; sAD: severe Alzheimer’s Disease; SoF: Sense of Familiarity

When looking at the SoF scale score, the LRT confirmed that the m2_recognition model (‘Type × Clinical status’ interaction, with ‘Artistic material’) significantly improved the model fit compared to m1_recognition (χ^2^(2) = 170.87, *P*  *<* 2.2e−16). The inclusion of ‘Artistic material’ significantly improved model fit, compared to the model without it (χ^2^ (1) = 9.6028, *P* = 0.001943), suggesting that recognition scores differed between musical and pictorial items. Therefore, following the principle of parsimony, m2_recognition model was retained for subsequent analyses. Pairwise comparisons with Tukey-correction of estimated marginal SoF scales scores in recognition session revealed significant differences between HC and both AD groups in targets (HC versus mAD: *z* = 3.954, *P* = 0.0002, *r* = 0.10; HC versus sAD: z = 6.10, *P* < 0.0001, *r* = 0.14; mAD versus sAD: *z* = 2.514, *P* = 0.032, *r* = 0.06) and in distractors discrimination (HC versus mAD: *z* = −4.425, *P* < 0.0001, *r* = 0.11; HC versus sAD: *z* = −5.407, *P* < 0.0001, *r* = 0.13). No significant differences were observed between mAD and sAD for distractors. However, all three groups significantly discriminated target from distractor items (HC: z = 17.873, *P* < 0.0001, *r* = 0.43; mAD: *z* = 16.903, *P* < 0.0001, *r* = 0.40; sAD: *z* = 14.383, *P*  *<* 0.0001*, r* = 0.34), reflecting preserved FBR across all clinical groups. Finally, pairwise comparisons within m2_recognition revealed higher SoF scales scores for pictorial items than musical items across all groups and item types (music versus painting: *z* = 3.339, *P* = 0.0008, *r* = 0.08). Together, these results show that, despite a gradient recognition performance consistent with disease severity, all clinical groups retained the ability to discriminate familiar from unfamiliar artistic items, indicating preserved FBR, even in the sAD group.

### MRI analyses

#### Whole-brain atrophy

Due to overwhelming anxiety, 1 mAD patient did not complete MRI examination. Therefore, 32 patients (17 mAD and 15 sAD participants) were included in subsequent analyses. The atrophy pattern of mAD compared with HC shows a significant decrease in GM volume in the bilateral hippocampus, in the left amygdala and in the right superior temporal pole (cluster-level, *P*_FWE_ < 0.05, *k* ≥ 60). Concerning sAD compared with HC, the progression of atrophy extends to new areas, such as the left amygdala (whose cluster includes the left hippocampus), the right middle and superior temporal gyrus, the right middle frontal gyrus, the left superior temporal gyrus, the right hippocampus, the right inferior parietal gyrus, the left precentral gyrus and the left middle temporal pole (cluster-level, *P*_FWE_ <0.05, *k* ≥ 60). Significant clusters are represented in Fig. 4 and full cluster statistics are reported in Table 3.

**Figure 4:**
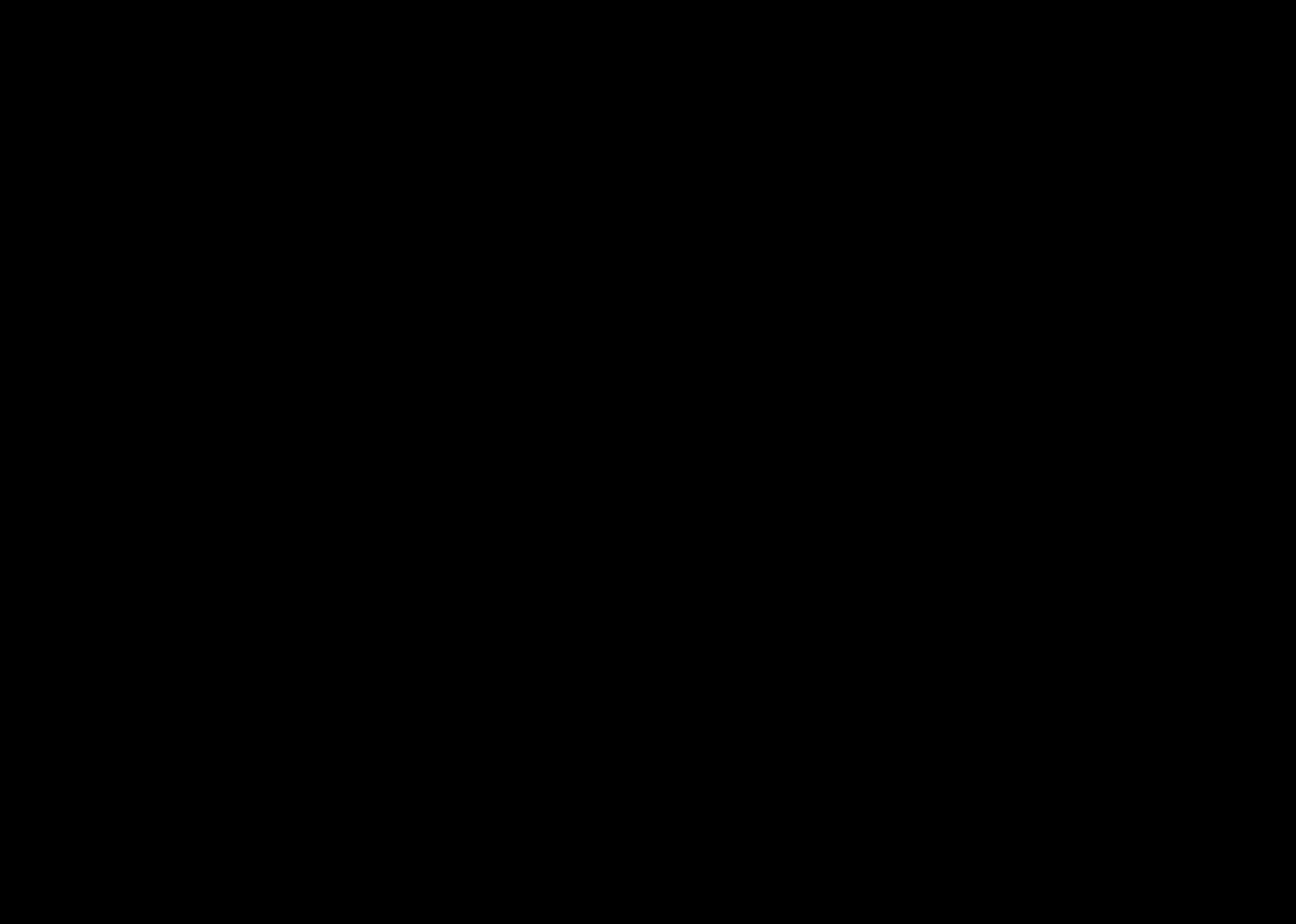
**Voxel-based morphometry representation of the cerebral atrophy in patient groups, compared to the HC group.** (**A**) mAD group (*n* = 17) versus HC (*n* = 24); (**B**) sAD group (*n* = 15) versus HC (*n* = 24). HC: healthy controls, mAD: moderate Alzheimer's disease, sAD: severe Alzheimer's disease. Results of the ANCOVA, including age, sex and total intracranial volume. Results are displayed at a voxel-level threshold of *P*_FWE_ < 0.05, with an extent threshold of *k* ≥ 60. Statistical maps are represented on an MNI template with 3D cortical render and 3 orthogonal slices (x = −27, y = 2, z = −14).

**Table 3:** Significant clusters of grey matter atrophy in Alzheimer’s disease patients compared with HC

AAL3 label	Cluster size (*k*)	MNI coordinates			*T*	Cluster-level *P*_FWE_
		x	y	z		
HC > mAD						
Right Hippocampus	1148	32	−22	−10	6.94	**<0**.001***
Left Amygdala	362	−22	2	−20	6.73	**<0**.001***
Right Superior Temporal Pole	347	40	15	−21	6.55	**<0**.001***
Left Hippocampus	147	−27	−22	−9	5.33	**<0**.001***
HC > sAD						
Left Amygdala	1108	−20	0	−18	7.53	**<0**.001***
*Including Left Hippocampus*						
Right Middle Temporal Gyrus	199	64	−21	−8	7.43	**<0**.001***
Right Superior Temporal Gyrus	403	45	−2	−10	7.28	**<0**.001***
Right Middle Frontal Gyrus	89	42	−3	57	7.24	**0**.001**
Left Superior Temporal Gyrus	104	−63	−24	8	6.74	**0**.001**
Right Hippocampus	653	32	−22	−10	6.59	**<0**.001***
Right Inferior Parietal Gyrus	104	−46	−51	48	5.95	**0**.001**
Left Precentral Gyrus	78	−26	−20	69	5.91	**0**.001**
Left Middle Temporal Pole	62	−44	16	−38	5.76	**0**.002**

Results are presented at a cluster-level family-wise error correction (*P*_FWE_ < 0.05, *k* ≥ 60 voxels). MNI coordinates (*x*, *y*, *z*) refer to the localization of the peak *T*-value, reported at voxel-level. Cluster volumes are shown. For brevity, each region that showed reduced grey matter is listed only once. When several peaks were observed in the same region, the coordinates refer to the localization of the highest *T*-value for that region. Regions were labelling using the AAL3 atlas. Significant results are displayed in bold font. **P < 0.01, ***P < 0.001.

#### Hippocampal atrophy

A focus on the hippocampal atrophy at the individual level reveals that the GM volume lost by both patient groups (mAD and sAD) ranges from 10.45% to 27.84% when compared to the HC group. Upon distinguishing between the left and right hippocampus, a range of relative atrophy was observed, with values ranging from 7.57% to 29.07% (left) and from 13.19% to 28.15% (right). Comparisons of global relative atrophy across clinical status revealed significant differences between HC and mAD (*χ*^2^ = −5.77, *P* = 2.31 × 10^−8^, *r* = 0.85) and between HC and sAD (*χ*^2^ = −4.75, *P* = 4.16 × 10^−6^, *r* = 0.82), but none between mAD and sAD. A significant discrepancy was observed for left and right hippocampus between HC and mAD (left: *χ*^2^ = −5.56, *P* = 8.07 × 10^−8^, *r* = 0.85; right: *χ*^2^ = −6.05, *P* = 4.40 × 10^−9^, *r* = 0.85) and between HC and sAD (left: *χ*^2^ = −4.93, *P* = 1.65 × 10^−6^, *r* = 0.80; right: *χ*^2^ = −4.39, *P* = 2.27 × 10^−5^, *r* = 0.81). Significant differences between the left and right hippocampus volume of GM have been detected for HC group (*W* = 0, *P* = 1.19 × 10^−7^, *r* = 0.87) and for sAD group (*W* = 3, *P* = 0.001, *r* = 0.82).

#### Multiple regression between the SoF and the grey matter volume

For music items, multiple regression showed a significant positive correlation between SoF scale score in recognition session and GM volume in the right caudate nucleus (peak MNI coordinates: *x* = 10, *y* = 9, *z* = −3, *T* = 6.28, *k* = 1566, cluster-level, *P*_FWE_  *<* 0.01, *k* ≥ 60). For pictorial items, no significant clusters were identified in patients’ group.

## Discussion

The main goal of our study was to demonstrate preserved FBR in patients with moderate to severe Alzheimer’s disease, for artistic items. During exposure sessions, although learning slopes differed significantly across groups (with patients requiring more sessions than HC), most patients (31 out of 33) successfully reached the learning criterion for at least one artistic material. In the recognition session, all three groups significantly discriminated target from distractor items, though discrimination performance showed a modest gradient consistent with disease severity. Taken together, these results indicate that FBR is preserved but modulated by disease severity. Structural MRI confirmed substantial hippocampal atrophy in both patients’ subgroups, rendering this preservation even more remarkable and underscoring the need to identify alternative neural substrates. Finally, regression analysis identified a positive association between GM volume in the right caudate nucleus and SoF score of recognition session for musical items.

The majority of Alzheimer’s disease patients successfully reached the established learning criterion for at least one artistic material. Specifically, the two patients who failed to meet the criterion belong to the mAD group and did not present the most severe neuropsychological profiles in our sample. More precisely, when looking at the performances in the different neuropsychological tests, it appears that both had MMSE scores above the mAD mean and CDR scores of 1, consistent with a moderate rather than severe disease stage. This observation, while limited by the small number of participants who did not meet the learning criterion, suggests that the degree of cognitive impairment alone may not reliably predict familiarity acquisition capacity. That failure to meet the criterion may instead reflect individual factors, such as anxiety or behavioural disturbances.

Learning slopes differed significantly across groups, with sAD patients requiring a longer temporal investment than mAD patients, and both patients’ groups more than HC, as evidenced by the gradual increase in the number of sessions over the course of the disease. However, progress was steady and positive across all clinical groups, reflecting a preserved but slowed familiarity acquisition process. Crucially, no significant effect of the artistic material on learning trajectories was observed, indicating that both music and paintings were equally effective in supporting SoF acquisition

Taken together, these results reveal a preserved capacity for familiarity acquisition, that paradigms relying on intentional encoding instructions or explicit ‘Yes/No’ recognition responses would likely have failed to detect in this population. Indeed, our approach was specifically designed to isolate familiarity processes, by bypassing attentional biases, analytical strategies and intentional encoding. In this context, using the SoF scale has allowed us to reliably capture the emergence of familiarity in a population with explicit memory impairment. In fact, patients’ ability to meet the learning criterion supports the hypothesis that this type of familiarity reflects automatic memory processes that are distinct from explicit episodic recollection or intentional learning.^62^ Our findings suggest that this implicit assessment is particularly pertinent in severe Alzheimer’s disease, where explicit memory is significantly disrupted. By leveraging a paradigm that favours unintentional encoding, our design aligns with current neurocognitive models positing that familiarity can be generated via perceptual or conceptual fluency, even in the absence of strategic and analytical processing.^63^

In the recognition session, all three clinical groups significantly discriminated target from distractor items, with high SoF scores for familiar items and low SoF scores for unfamiliar items. Although discrimination performance showed a modest but significant attenuation, consistent with disease severity, our results showed the preservation of the SoF across all groups, including sAD. Our findings stand in apparent contrast with the prevailing view in the existing literature, which holds that FBR is compromised from the earliest stages of Alzheimer’s disease.^29,30^ According to the dual-process model, recognition can be based on two distinct mechanisms: familiarity and recollection.^24,26^ Numerous studies demonstrate that familiarity is underpinned by different structures of the medial temporal lobe, such as perirhinal and parahippocampal cortices. For instance, Bowles *et al.,* documented a case of resection of the left anterior temporal lobe, encompassing the perirhinal and entorhinal cortex, while preserving the hippocampus. When the subject was submitted to the Remember/Know Procedure, the results demonstrated a severe familiarity deficit, without any recollection impairments.^64^ Furthermore, research has demonstrated that damage to the left parahippocampal gyrus is associated with familiarity deficits, while a reduction in hippocampal volume is associated with recollection disorders.^65^ Given that the perirhinal cortex is among the earliest structures to be degraded in Alzheimer’s disease, these findings would predict severely impaired SoF even at mild stages. Yet our patients, despite confirmed significant hippocampal atrophy and the broader medial temporal lobe degeneration evidenced by whole-brain VBM, demonstrated preserved familiarity acquisition of artistic items.

One explanation lies in the nature of the stimuli and the encoding conditions used in our paradigm. Unlike verbal material, artistic stimuli (most notably music and painting) are better encoded and later recognized in severely amnesic Alzheimer’s disease, suggesting that such material may more effectively support recognition processes than verbal items.^37^ Processing music does not require explicit analytical strategies, in contrast to verbal items, which require active semantic analysis making them more difficult to recognize. Moreover, musical listening elicits unconscious cerebral processing by extracting tonal structure, rhythm and timbre. This automatic extraction of musical structure may engage subcortical networks independently of explicit memory systems. Repeated exposure may have strengthened implicit perceptual traces via these alternative networks, promoting the development of FBR, independently of the perirhinal integrity. This explanation is supported by various studies indicating that listening to music activates the salience network and dopaminergic circuits in patients with Alzheimer’s disease, thereby transiently improving network connectivity. Furthermore, the absence of a significant effect of artistic material on learning trajectories suggests that music and paintings engaged these preserved mechanisms to an equivalent extent. By contrast, SoF scale scores in recognition session for paintings are slightly higher than those obtained for music. Given the small effect sizes reported, it is likely that this difference reflects a greater perceptual distinction between targets and distractors rather than a true superiority of pictorial memory. This constitutes a genuine methodological limitation to be considered in future studies.

From a neuroanatomical perspective, the patients in our sample demonstrated a reduction in hippocampal volume ranging from 10.45% to 27.84%, compared to the HC group, consistent with previously reported values. Mueller *et al.*^66^ reported an average hippocampal atrophy of 15.9% in Alzheimer’s disease patients compared to HC, with an annual rate of 4.4% in patients versus 1.7% in HC groups.^67^ Hemispheric comparisons revealed significant asymmetry between left and right hippocampi in HC and sAD groups, but not in mAD. In healthy adults, this rightward hippocampal asymmetry is well-documented in the extant literature, with a larger right hippocampus.^68^ In sAD, the observed asymmetry may be indicative of a predominance of left hippocampal atrophy at advanced disease stages, consistent with evidence that this asymmetry becomes more pronounced as the disease progresses.^66,69^ The predominance of left hippocampal atrophy observed in sAD is consistent with evidence suggesting a leftward dominance for familiarity processes,^27^ although direct evidence of functional lateralization of familiarity for artistic items remains limited. Finally, the absence of significant asymmetry in mAD may reflect a transitional stage or a reduced statistical power in this subgroup.

Beyond confirming the extent of hippocampal atrophy, we examined whether GM volume was positively associated with SoF scale score in recognition session across patients, controlling for age, sex, TIV and global cognitive severity (using the MMSE). This analysis revealed a positive correlation between GM volume and SoF scale score in recognition session, specifically for musical items in the right caudate nucleus, surviving cluster-level FWE corrections. No significant association was found for pictorial items. This finding is consistent with the established role of the caudate nucleus in reward-based behavioural learning, information selection, and implicit procedural memory.^70-73^ In music neuroscience, the caudate has been specifically linked to the anticipation of musical pleasure and reward signalling during exposure to familiar excerpts.^74^ Our findings suggest that the caudate may contribute to the integration of familiarity signals. In Remember/Know paradigms, Know responses are associated with increased caudate activity, reflecting implicit memory strength that informs recognition decisions.^25^ Accordingly, artistic familiarity may rely on reward and implicit learning networks to compensate for hippocampal impairment, with the caudate playing a role in distinguishing between ‘familiar’ and ‘unfamiliar’ stimuli. This mechanism is particularly relevant for emotionally salient artistic items such as music. Indeed, in Alzheimer’s disease, musical experiences engage salience and dopaminergic networks within the dorsal striatum, including the caudate.^43^ A transient augmentation of this network, facilitated by musical stimulation, has been associated with enhanced well-being in patients. Notably, the caudate is relatively preserved until later stages.^32^ Furthermore, Grahn and Rowe demonstrated that the perception of musical beats engages the caudate nucleus, without any conscious motor intention,^75^ suggesting that repeated exposure to musical excerpts may have promoted the encoding of an implicit sensory-motor representation, contributing to FBR independently of explicit memory retrieval. The specificity of this finding to musical items is consistent with the role of the caudate nucleus in musical memory and reward processing. By contrast, pictorial memory engages regions distributed along the ventral cortical pathway, including early visual cortex, inferior temporal cortex, lateral occipital cortex, fusiform gyrus and superior temporal sulcus.^76^ These regions are well known to be more severely affected by neurodegeneration in Alzheimer’s disease,^3^ leading to reducing detectable structural variance in a VBM regression approach. Together, these neuroimaging findings suggest that residual FBR in Alzheimer’s disease may rely on reward and implicit learning networks that remain relatively preserved, motivating future task-based fMRI studies.

This design enhances ecological validity compared with conventional Alzheimer’s disease paradigms, typically based on abstract or decontextualized stimuli. Indeed, a tailored encoding procedure was specifically developed for patients with severe Alzheimer’s disease, allowing non-analytic and implicit learning strategies to be engaged. Importantly, participants were not instructed to intentionally encode the items, thereby avoiding analytic strategies known to modulate mere exposure effects and FBR.^63^

Although the SoF scale is not a gold standard in the literature, it offers several advantages over classical Remember/Know^26^ or Old/New Procedures.^24^ Indeed, behavioural cues were used to quantify familiarity in contexts where language impairment may manifest.^77^ Furthermore, the SoF scale provided a graded continuum, where familiarity and recollection represent merely initial stages in the process of capturing this dynamic. This approach confers genuine clinical pertinence and supports its use as an ecological assessment tool. Finally, to prioritize SoF over recollection, responses four to six were collapsed, at the cost of reduced granularity in memory profiling. A data-driven classification stratified patients into two subgroups, maximizing clinical relevance and ecological validity of the results obtained. This empirical approach avoided a priori assumptions and thresholds, to ensure the intra-group validity of the findings. It used five neuropsychological scores to ensure multidimensional neuropsychological coherence. Nonetheless, the limited sample (*N* = 33) may risk overfitting and limit the generalizability. Furthermore, the cerebral volume was not considered, but this could be incorporated in future external validations. Finally, a notable strength of the present study is the ability to retain 33 patients with Alzheimer’s disease across multiple test sessions and MRI examinations. This level of feasibility is uncommon in this population, which reinforces the robustness of the behavioural findings. Furthermore, the emotional state of the participants was not directly controlled during exposure and recognition sessions. Given that patients with Alzheimer’s disease frequently experience depression, anhedonia or apathy,^78^ individual differences in hedonic responses to artistic stimuli may have introduced variability in SoF scale scores. To mitigate this potential confounding factor, participants with significant depressive symptoms were excluded, based on their GDS score. Nevertheless, residual variability in emotional responsiveness cannot be ruled out and may have influenced interindividual differences in learning speed or recognition performance. Future studies should systematically assess hedonic responses to artistic stimuli to disentangle the respective contributions of emotional arousal and familiarity-based mechanisms to familiarity acquisition.

In summary, this study provides a multi-systemic perspective on memory in Alzheimer’s disease, highlighting the persistence of specific forms of implicit long-term encoding. While research often focuses on memory impairments, spared functions remain poorly understood. This study has contributed to better understand recollection and familiarity processes in Alzheimer’s disease, along with the distinction between explicit and implicit memory. It demonstrates clinical value by reactivating emotional responses linked to past experiences, even in the absence of episodic recovery, with potential benefits for patients’ emotional well-being and behaviour. Consequently, art emerged as a privileged medium for emotionally modulated implicit learning. The dual artistic modality enabled the assessment of the multimodality of familiarity and the specific vulnerability of different sensory networks. By integrating neuroanatomy and brain atrophy assessment, we extend beyond the scope of conventional behavioural analysis, and we strengthen our behavioural findings. Future fMRI studies could identify regions activated during item recognition and their relation to SoF. Overall, even in advanced Alzheimer’s disease, residual long-term memory mechanisms remain accessible, particularly through artistic stimuli, opening promising perspectives for art-based interventions.

## Supplementary Material

fcag399_Supplementary_Data

## Data Availability

The data that support the findings of this study are available from the corresponding author, upon reasonable request. The R code used for statistical analyses is publicly available at https://github.com/cvesque/INCAS_Behavioral-Analysis
