# Supplementary material for: Newly learned items form long-term memory in severe Alzheimer’s disease despite hippocampal atrophy
Source: Brain Commun. 2026 Oct 21;8(6):fcag399. doi: 10.1093/braincomms/fcag399 (PMC13748755; doi:10.1093/braincomms/fcag399)
Supplement: Supplementary file 1 [file fcag399_supplementary_data.docx]

## Materials & Methods

To accurately reflect disease progression, we performed a data-driven stratification of Alzheimer’s disease patients into subgroups, according to the severity of their symptoms. Hierarchical clustering was conducted on five neuropsychological tests (i.e. CDR, MMSE, BEC96, Five-Words, DO80), using Ward’s method with Euclidean distance. Prior to clustering, all scores were normalized (z-scores) to facilitate meaningful comparisons. A dendrogram was generated to assess the general structure of the data visually. To provide quantitative support for this visual assessment, the optimal number of clusters was determined using the *NbClust* package in *RStudio*, which aggregates 30 clustering validity indices (Charrad *et al.,* 2014). Based on this analysis, a non-hierarchical k-means cluster analysis was performed, with the number of clusters fixed by the previous analysis.

## Results

The hierarchical clustering dendrogram revealed two main clusters. Among the 30 validity indices tested, 12 suggested a 2-cluster solution, supporting our initial choice based on visual inspection of the dendrogram (Supplementary Fig. 1). The data-driven clustering procedure yielded two groups of Alzheimer's disease: moderate Alzheimer’s disease (mAD, *N* = 18) and severe Alzheimer's disease (sAD, *N* = 15).

Supplementary Fig. 1 Data-driven clustering of Alzheimer’s disease patients. (A) Hierarchical clustering dendrogram based on score on five neuropsychological tests (CDR, MMSE, BEC96, Five-Words, DO80). This analysis was performed using Ward's method (‘Ward.D2’) with Euclidean distance and the dendrogram revealed two main clusters with Alzheimer’s disease patients (n = 33); (B) Number of clusters suggested by the 30 validity indices of the *NbClust RStudio* package; 12 out of 30 indices supported the 2-cluster solution. This procedure yielded two groups: moderate Alzheimer’s Disease (mAD; n=18) and severe Alzheimer’s Disease (sAD; n=15). CDR: Clinical Dementia Rating, MMSE: Mini-Mental State Examination, BEC96: cognitive battery test, DO80: 80-images oral naming test.

A

B

## Reference

Charrad, M., Ghazzali, N., Boiteau, V., & Niknafs, A. (2014). NbClust : An R Package for Determining the Relevant Number of Clusters in a Data Set. *Journal of Statistical Software*, *61*(6), 1‑36. https://doi.org/10.18637/jss.v061.i06
